# The marine conservation deposits of Monte San Giorgio (Switzerland, Italy): the prototype of Triassic black shale Lagerstätten

**DOI:** 10.1186/s13358-024-00308-7

**Published:** 2024-03-04

**Authors:** Christian Klug, Stephan N. F. Spiekman, Dylan Bastiaans, Beat Scheffold, Torsten M. Scheyer

**Affiliations:** 1 Universität Zürich, Paläontologisches Institut, Karl-Schmid-Strasse 4, 8006 Zurich, Switzerland; 2 Staatliches Museum für Naturkunde Stuttgart, 70191 Stuttgart, Germany

**Keywords:** Konservat-Lagerstätten, Taphonomy, Marine reptiles, Exceptional preservation

## Abstract

Marine conservation deposits (‘Konservat-Lagerstätten’) are characterized by their mode of fossil preservation, faunal composition and sedimentary facies. Here, we review these characteristics with respect to the famous conservation deposit of the Besano Formation (formerly Grenzbitumenzone; including the Anisian–Ladinian boundary), and the successively younger fossil-bearing units Cava inferiore, Cava superiore, Cassina beds and the Kalkschieferzone of Monte San Giorgio (Switzerland and Italy). We compare these units to a selection of important black shale-type Lagerstätten of the global Phanerozoic plus the Ediacaran in order to detect commonalities in their facies, genesis, and fossil content using principal component and hierarchical cluster analyses. Further, we put the Monte San Giorgio type Fossillagerstätten into the context of other comparable Triassic deposits worldwide based on their fossil content. The results of the principal component and cluster analyses allow a subdivision of the 45 analysed Lagerstätten into four groups, for which we suggest the use of the corresponding pioneering localities: Burgess type for the early Palaeozoic black shales, Monte San Giorgio type for the Triassic black shales, Holzmaden type for the pyrite-rich black shales and Solnhofen type for platy limestones.

## Introduction

**Fig. 1:**
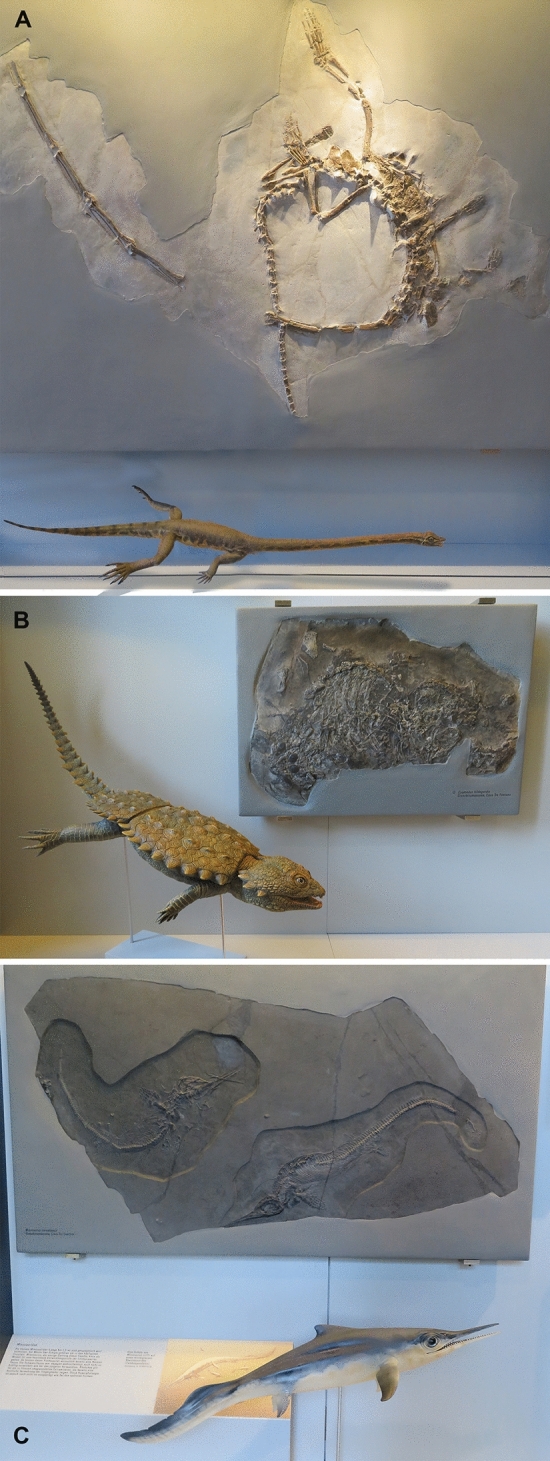
Three important marine reptiles from the Middle Triassic of Monte San Giorgio with their reconstructions, recently crafted by Beat Scheffold. **A** *Tanystropheus*, PIMUZ T 2817. **B** *Cyamodus*, PIMUZ T58. C, *Mixosaurus*, PIMUZ T 4923 (top) and PIMUZ T 4376 (bottom)

**Fig. 2:**
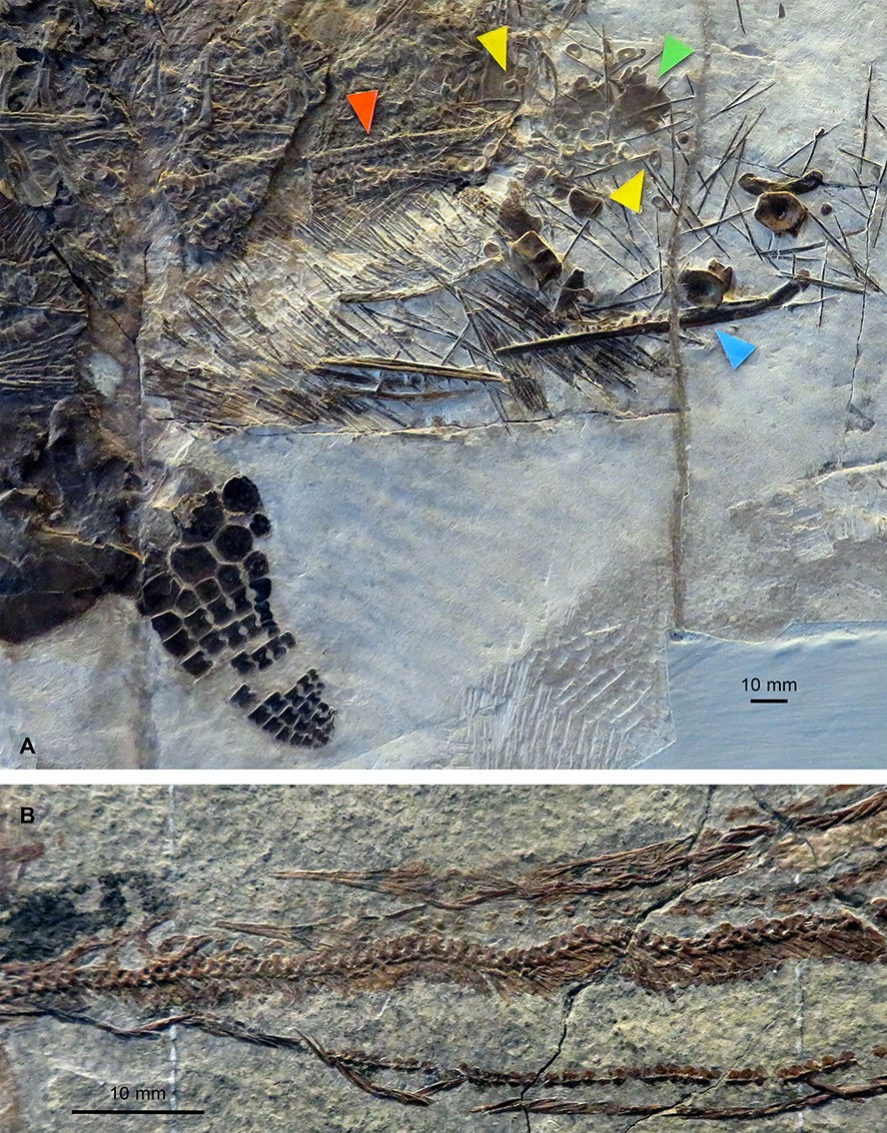
Fossilized foetuses inside the mother, examples from the Middle Triassic of Monte San Giorgio. **A** *Mixosaurus*, PIMUZ T 4830 (e.g., Brinkmann, 1996; Miedema et al., 2023). **B** *Saurichthys*, PIMUZ T 3917 (e.g., Maxwell et al., 2018)

Since the pioneering work of Bernhard Peyer, who began his excavations in the Swiss canton of Ticino in 1924 (Lanz & Felber, 2020; Peyer, 1934), many books and articles have appeared about the conservation deposit of the Middle Triassic of Monte San Giorgio (for the dating of the section see Furrer et al., 2008); the latest comprehensive works are the volumes by Olivier Rieppel (2019) and by Heinz Lanz and Markus Felber (2020). Classically, the monographs (mostly published in the ‘Schweizerische Paläontologische Abhandlungen’, now the *Swiss Journal of Palaeontology*) on the vertebrates of this locality comprised the most important pioneering work. The rich fauna of fossil vertebrates provides a unique insight into the recovery of shallow marine ecosystems following the devastation of the largest mass extinction event in Earth history, the Permian–Triassic boundary mass extinction (Chen & Benton, 2012; Scheyer et al., 2014a). This fauna includes a broad array of remarkable marine reptiles (Figs. 1, 2), for which this Lagerstätte is arguably best known (e.g., Bürgin et al., 1989; Rieppel, 2019). In particular, the fascinating reptile *Tanystropheus* contributed to the fame of the Besano Formation (Fig. 1A). Originally known from fragmentary remains, mostly comprising highly elongated and hollow bones, these remarkable yet poorly understood remains led researchers to erroneous ideas about their systematic assignment (e.g., v. Meyer 1847–1855; Nopsca, 1923; Edinger, 1924). Only with the discovery of the spectacular skeletons excavated by Peyer at Monte San Giorgio did their correct identification become a possibility. Today, these elongate bones are known to be hyper-elongated cervical vertebrae that belonged to an extremely long necked, likely aquatic to semi-aquatic archosauromorph reptile (e.g., Beardmore & Furrer, 2017; Jaquier & Scheyer, 2017; Kuhn-Schnyder, 1959; Nosotti, 2007; Peyer, 1930, 1931a; Renesto, 2005; Spiekman & Mujal, 2023; Spiekman & Scheyer, 2019; Spiekman et al., 2020a, 2020b; Tschanz, 1986, 1988; Wild, 1973, 1980). Its terrestrial relative *Macrocnemus* was also studied by various authors (Herbst et al., 2021; Jaquier et al., 2017; Kuhn-Schnyder, 1962; Miedema et al., 2020; Nopcsa, 1930; Peyer, 1937; Premru, 1991; Renesto & Avanzini, 2002; Rieppel, 1989a; Saller, 2016).

**Fig. 3:**
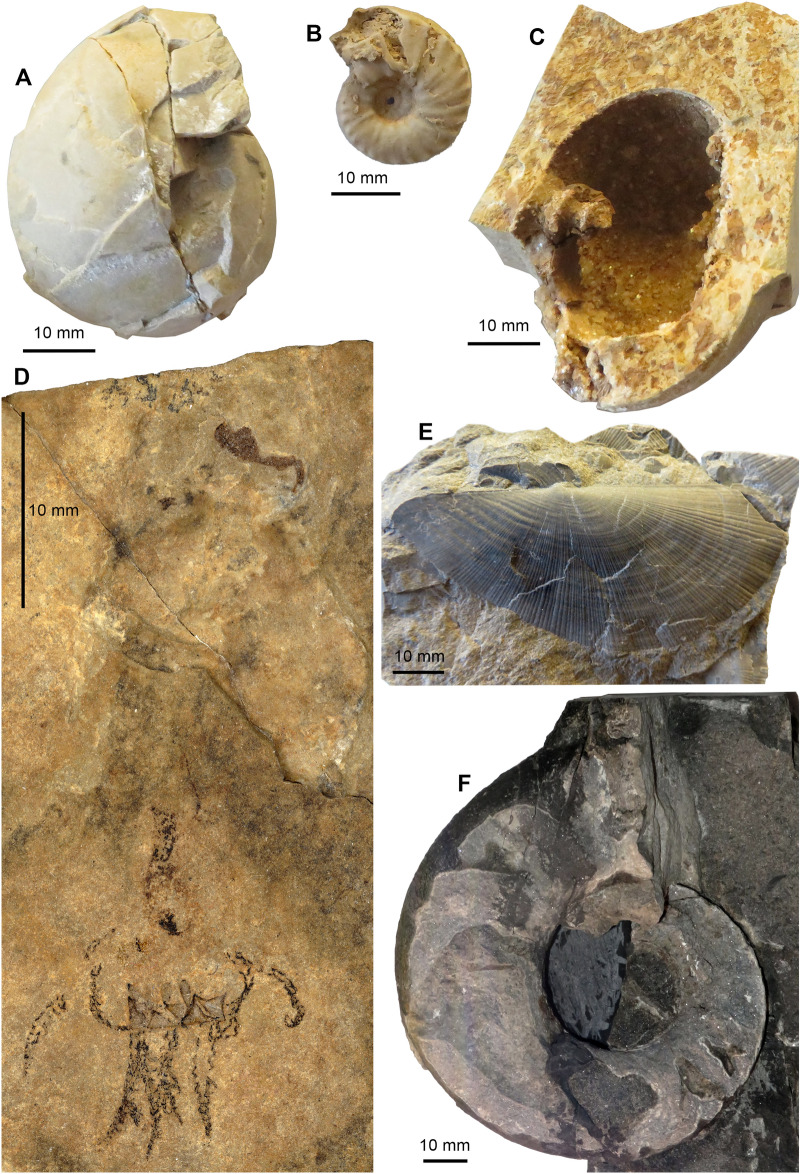
Fossilized mollusks from the Middle Triassic of Monte San Giorgio (see Rieber, 1969, 1970, 1973; Pieroni, 2022). A, *Proarcestes extralabiatus*, internal mould. B, *Repossia acutenodosa*, silicified internal mould. C, *Proarcestes extralabiatus*, external mould. D, *Phragmoteuthis ticinensis* with complete arm crown, cephalic cartilage, oesophagus and ink sac. E, *Daonella caudata*. F, *Pleuronautilus* sp., internal mould

To date, the fossil localities of Monte San Giorgio still represent the best-studied record of a Middle Triassic marine ecosystem worldwide, rich in marine reptiles, fishes, invertebrates, and plants (Fig. 3), which led to its recognition as a UNESCO World Heritage site in 2003 (Felber et al., 2004; https://whc.unesco.org/uploads/nominations/1090.pdf). Despite its long history, the relevance of the site is still increasing, both through continuing studies of the historical collections, and through comparisons to new abundant fossil material, especially also of marine reptiles, from contemporaneous sites on the eastern Tethys margin in what is now southern China (e.g., Benton et al., 2014).

**Fig. 4:**
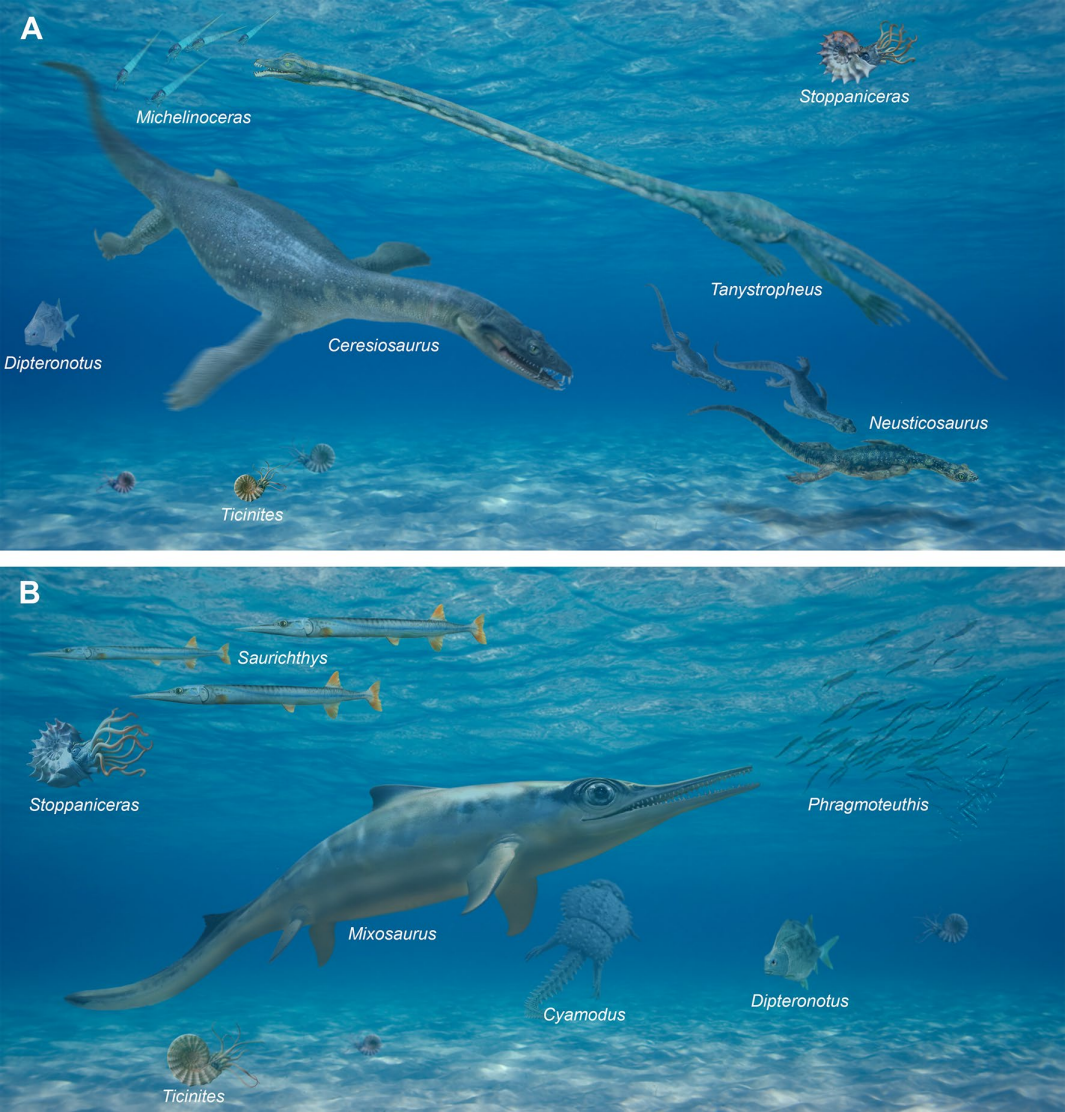
Reconstructions of some animals from Monte San Giorgio by Beat Scheffold. Note that not all of the depicted taxa may have co-occurred in time or in space (habitat depth, etc.). At Monte San Giorgio, the water depth was likely greater then shown in these images. **A** Meride Limestone (Ladinian). **B** Besano Formation (Anisian)

**Fig. 5:**
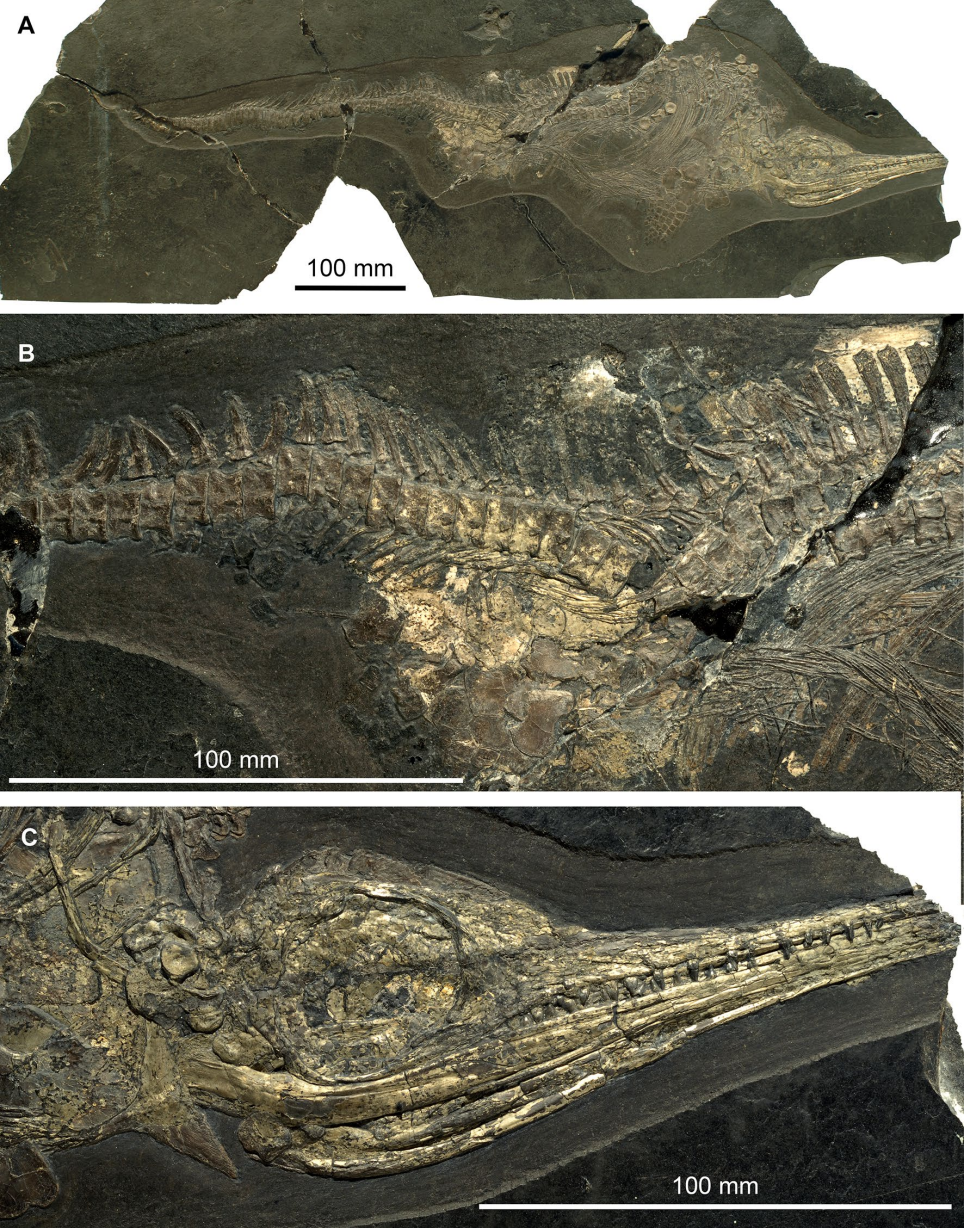
Exceptionally preserved ichthyosaur *Mixosaurus cornalianus* (Bassani, 1886). PIMUZ T 1839, Besano Formation, Monte San Giorgio (**A**). The skull of the specimen (**C**) is covered by a thin pyrite crust and in the pelvic region (**B**), some phosphatized soft-tissue remains are discernible. *Mixosaurus* belongs together with *Neusticosaurus* to the most common reptile fossils of the Besano Formation. This specimen was excavated in 1931 and prepared in 2019 by Christian Obrist

Besides *Tanystropheus*, there are of course other vertebrate fossils of scientific importance known from Monte San Giorgio (Figs. 1, 2, 4). These comprise, for example, the following vertebrate groups (for a more complete bibliography, see Albisetti & Furrer, 2022):

- The rauisuchian *Ticinosuchus* (e.g., Krebs, 1965; Lautenschlager & Desojo, 2011; Nesbitt, 2011; Pinna & Arduini, 1978);
- Placodonts (e.g., Kuhn, 1942; Kuhn-Schnyder, 1960; Neenan et al., 2014; Peyer, 1931b, 1931d; Pinna, 1992; Scheyer, 2010);
- Eosauropterygians (e.g., Beardmore & Furrer, 2016; Carroll & Gaskill, 1985; Cornalia, 1854; Hänni, 2004; Hugi, 2011; Hugi & Scheyer, 2012; Hugi et al., 2011; Kuhn-Schnyder, 1966, 1967, 1987; Mariani, 1923; Nosotti & Rieppel, 2003; Peyer, 1931c; Renesto, 1993; Rieppel, 1989b; Sander, 1988, 1989b);
- Thalattosaurs (e.g., Bastiaans et al., 2023a, 2023b; Klein et al., 2023; Kuhn, 1946a, 1946b, 1946c; Kuhn-Schnyder, 1988; Müller, 2005; Nopcsa, 1925; Peyer, 1936a, 1936b; Rieppel, 1987; Rieppel et al., 2005);
- Ichthyosaurs (e.g., Sander, 1989a; Brinkmann, 1996, 1998, 1999, 2004; Dal Sasso & Pinna, 1996; Maisch & Matzke, 1997, 1998; Maisch et al., 2006; Kolb et al., 2011; Pardo-Pérez et al., 2020; Renesto et al., 2020; Bindellini et al., 2021; Miedema et al., 2023), a recently prepared specimen is shown in Fig. 5;
- Chondrichthyans (e.g., Kuhn, 1946a; Mutter, 1998a, 1998b; Rieppel, 1981, 1982);
- Actinopterygians (e.g., Argyriou et al., 2016; Bürgin, 1990a, 1990b, 1992, 1995, 1999a, 1999b; Guttormsen, 1937; Kuhn, 1946b; Lombardo, 1999, 2013; Lombardo & Tintori, 2004; Lombardo et al., 2012; López-Arbarello et al., 2014, 2016, 2019; Maxwell et al., 2013, 2015; Mutter, 2001, 2004; Mutter & Herzog, 2004; Rieppel, 1985a, 1985b; Romano & Brinkmann, 2009; Scheyer et al., 2014a, 2014b; Schwarz, 1970; Tintori, 1990; Wilson et al., 2013);
- Sarcopterygians (e.g., Cavin et al., 2013, 2017a, 2017b; Ferrante & Cavin, 2023; Renesto & Stockar, 2018; Rieppel, 1980, 1985a, 1985b).

These materials gain increasing attention because of their usefulness for comparisons with related taxa from other Triassic localities. Especially in China, several localities have produced articulated skeletons, mostly of vertebrates, that resemble the Monte San Giorgio specimens not only morphologically, but also with respect to preservation and facies of the sedimentary matrix (e.g., Hu et al., 2011; Jiang et al., 2005, 2009, 2020; Li, 2006; Lu et al., 2018; Rieppel et al., 2000; Sun et al., 2016; Wang et al., 2008). This has revealed that there are numerous Triassic localities worldwide with more or less similar depositional conditions and thus fossil preservation as at Monte San Giorgio. Consequently, Monte San Giorgio has become a reference in research on this type of conservation deposits (cf. Arif et al., 2019; Benton et al., 2013, 2022; Frey et al., 2019; Seilacher, 1970, 1990; Seilacher et al., 1985).

Accordingly, we briefly outline the properties of the Besano Formation, Cava inferiore, Cava superiore, Cassina beds and the Kalkschieferzone of Monte San Giorgio. We compare these to those of other Triassic conservation deposits worldwide. For this purpose, we characterized the six Swiss Triassic conservation deposits and 39 other globally important Phanerozoic Lagerstätten including Ediacara using various sedimentological and palaeontological traits. These were then evaluated using multivariate analyses. The aims were (1) to identify similarities and differences of these Lagerstätten; (2) to identify the palaeoecological conditions in order (3) to better understand under which circumstances the various types of Lagerstätten have formed.

## Methods

**Fig. 6:**
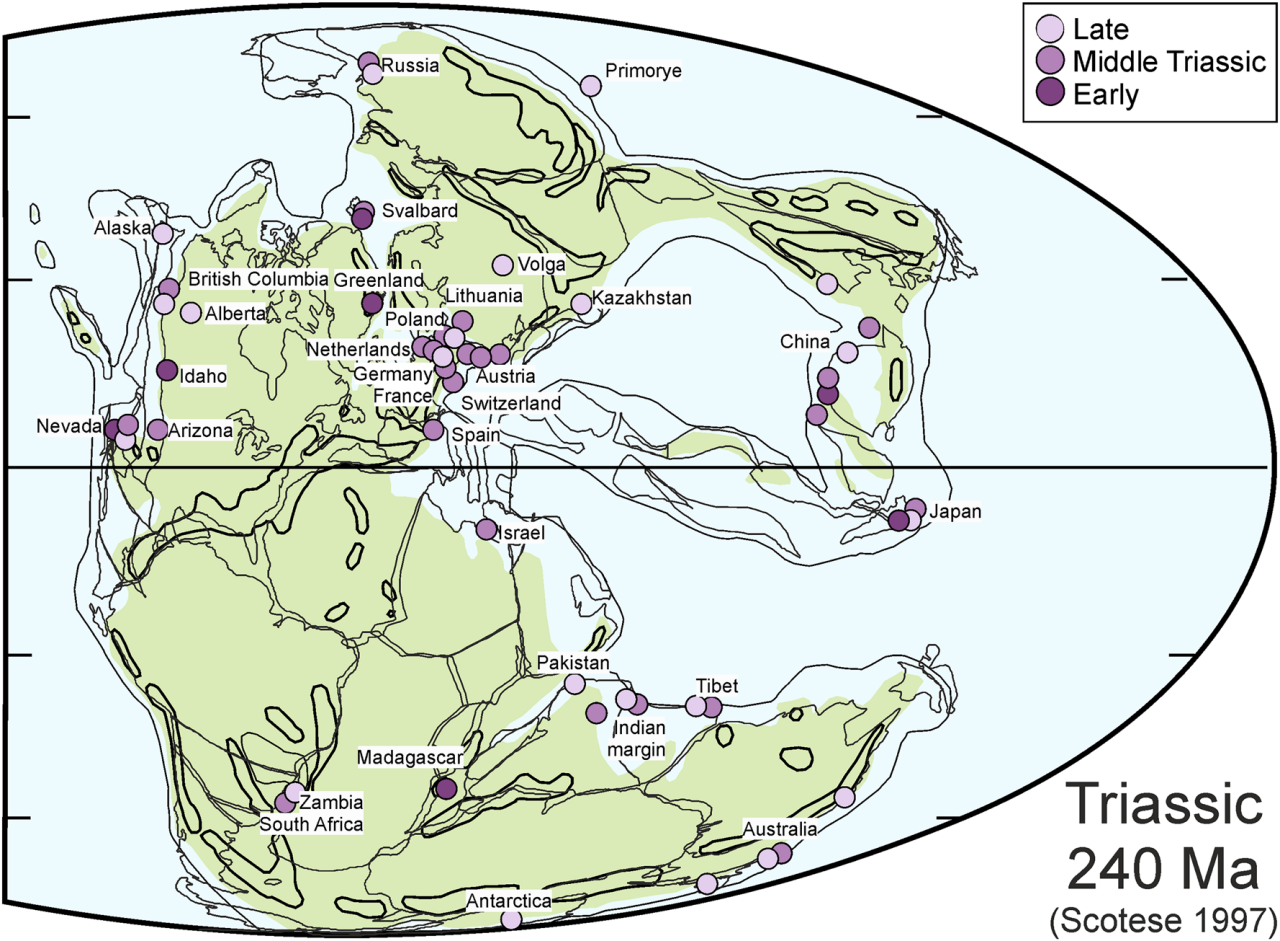
Palaeogeographic map (modified after Scotese, 1997) with data from Brinkmann et al. (2010) and Benton et al. (2013). Note that most of the conservation deposits included here lie in tropical or moderate latitudes, while occurrences of disarticulated materials are also known from boreal to arctic regions (not differentiated here)

Here, we follow the approach of Frey et al. (2019) in using the questionnaire published by Seilacher et al. (1985) to compare marine conservation deposits starting with the Ediacaran through a variance–covariance principal component analysis. The variables included information (often absence/presence) on marine basin size, sedimentary facies, palaeolatitude (Fig. 6), thickness of the succession, duration of the time interval in which the exceptionally preserved fossils formed, sea level, sediment structures, pyrite in sediment, faunal composition, trace fossils, infauna, epibenthos, pelagic macrofossils, death marches, landing marks, soft parts, cuticles, roll marks, current alignment, aragonite, internal moulds (pyritic or other), concretions, deformation, carbonization, soft part preservation mode (phosphatization, pyritization, silicification or in clay minerals), and preservation by obrution, stagnation or microbial mats. For the analyses, we omitted ‘life position’, because this was uniformly coded as 0. The original dataset of Frey et al. (2019) included only 21 Lagerstätten.

For this study, we added data of 34 localities to allow a global comparison of Triassic conservation deposits, which represents an incomplete but representative sample of known Lagerstätten (Tables 1, 2). Our list is not intended to be all inclusive for global Lagerstätten but was assembled with the intention of assessing the characteristics of the Triassic Monte San Giorgio in a global framework. For the Early Triassic, we included the Paris biota (Thaynes Fm) from Idaho, USA (Brayard et al., 2017; Doguzhaeva et al., 2018), the fauna from Kap Stosch, Greenland (Wordie Creek Fm; Brinkmann et al., 2010; Kear et al., 2016), the famous fish nodules from the Middle Sakamena Formation of Madagascar (Beltan, 1996; Brinkmann et al., 2010; Kogan & Romano, 2016), the ichthyosaur- and thalattosaur-bearing faunas of the Sulphur Mountain Formation from British Columbia and Alberta, Canada (Bastiaans et al., 2023a, 2023b; Neuman, 1992, 2015) and of the Vikinghøgda Formation in Svalbard (Hurum et al., 2018).

**Table 1:** Coding for the comparison of conservation deposits based on characteristics supposed by Seilacher et al. (1985), using data and modifications by Frey et al. (2019)

Name	Country, region	Age (not coded)	Marine basin size	Facies	Palaeolatitude	Thickness	Duration	Sea level	Sediment structures	Pyrite	Fauna	Traces	Infauna	Epibenthos	Pelagics (macro)
Ediacara	Australia	Proterozoic	1	1	1	1	1	0	1	0	0	0	0	1	0
Maotianshan Shales	S China	Cambrian	1	1	0	1	0.5	0.5	1	1	0	0	0	1	1
Burgess Shale	Canada	Cambrian	1	1	0	1	0.5	1	0	0	0	0	1	1	1
Fezouata Fm	Morocco	Ordovician	1	1	1	1	1	0.5	1	1	0	0	0	1	1
Hunsrück Shale	Germany	Devonian	1	1	0	1	0.5	1	0	1	0	1	0	1	0
Gogo Fm	Australia	Devonian	1	1	0	1	0.5	0.5	0	0	1	0	0	1	1
Thylacoceph. Layer	Morocco	Devonian	1	1	1	0.5	0.5	1	0	1	1	1	0	1	0
Madene HBS	Morocco	Devonian	1	1	1	0	0	0.5	0	0	0	1	0	1	0
Bear Gulch Lst	Montana	Carboniferous	1	0	0	1	0.5	0	0	0	1	0	0	1	0
Francis Creek Shale	Illinois	Carboniferous	1	1	0	0.5	0.5	0	0	1	0	1	0	1	0
Thaynes Gp	Nevada	Early Triassic	1	1	0	1	0	0.5	0	1	0	0	0	1	1
Wordie Creek Fm	Greenland	Early Triassic	1	1	1	1	0.5	0.5	0	0	0	0	0	1	1
Sakamena Gp	Madagascar	Early Triassic	1	1	1	0.5	0.5	0.5	0	0	1	0	0	0	1
Candelaria Fm	Nevada	Early Triassic	1	1	0	1	0	0.5	0	0	0	0	0	1	0
Osawa Fm	Japan	Early Triassic	1	1	0	1	0.5	1	0	0	0	0	0	1	1
Nanlinghu Fm	S China	Early Triassic	1	0	0	1	0.5	0.5	0	1	1	0	0	0	1
Jialingjiang Fm	S China	Early Triassic	1	0	0	1	0	0.5	0	0	1	0	0	0	1
Sulphur Mt Fm	Alberta, Can	Early Triassic?	1	1	0	1	0.5	0.5	0	0	1	0	0	1	1
Vikinghøgda Fm	Svalbard	Early Triassic?	1	1	1	1	0.5	0.5	0	0	0	1	0.5	1	1
Besano Fm	Switzerl	Mid Triassic	1	1	0	1	0.5	0.5	0	1	1	0.5	0	0	1
Cava inferiore	Switzerl	Mid Triassic	1	0	0	0	0	0.5	0	0	1	0	0	0	1
Cava superiore	Switzerl	Mid Triassic	1	0	0	0.5	0	0.5	0	0	1	0	0	0	1
Cassina	Switzerl	Mid Triassic	1	0	0	0	0	0.5	0	1	1	0	0	0	1
Kalkschieferz	Switzerl	Mid Triassic	1	0	0	1	0	0.5	0	0	0	0.5	0	0.5	1
Prosanto Fm	Switzerl	Mid Triassic	1	1	0	1	0.5	0.5	0	0	1	0	0	1	1
Partnach Fm	Austria	Mid Triassic	1	0	0	0.5	0.5	0.5	0	0	1	0	0	1	0
Prida Fm	Nevada	Mid Triassic	1	0	0	1	0.5	0.5	0	0	0	0	0	1	1
Botneheia Fm	Svalbard	Mid Triassic	1	1	1	1	0.5	0.5	0	1	1	0	0	1	1
Guanling Fm Luop	S China	Mid Triassic	1	0	0	1	0	0.5	0	1	0	0	0	1	1
Guanling Fm Panx	S China	Mid Triassic	1	0	0	0.5	0	0.5	0	0	1	0	0	0	0
Falang Fm Xingyi	S China	Mid Triassic	1	0	0	0.5	0.5	0.5	0	0	1	0	0	0	0
Xiaowa Fm	S China	Late Triassic	1	0	0	0.5	0	0.5	0	0	1	0	0	1	1
Calcare di Zorzino	Italy	Late Triassic	1	0	0	0.5	0	1	0	1	1	0	0	1	1
Lunz Fm	Austria	Late Triassic	1	1	0	0.5	0	0.5	0	1	0	0	0	1	1
Seefeld Mb	Austria	Late Triassic	1	0	0	0.5	0	0.5	0	1	1	0	0	0	1
Psilonotenton Fm	Germany	Jurassic	0	1	0	0	0	0	1	0	0	0	0	1	0
Posidoniensch. Fm	Germany	Jurassic	1	1	0	1	0.5	0.5	0	1	1	1	0	1	0
Oxford Clay Fm	UK	Jurassic	1	1	0	0.5	0.5	1	0	1	0	0	0	1	0
Terre noires	France	Jurassic	1	1	0	0.5	0.5	0.5	0	1	0	0	0	1	0
Altmühltal Fm	Germany	Jurassic	1	0	0	1	0.5	0.5	0	0	1	1	0	1	0
Painten Fm	Germany	Jurassic	0.5	0	0	1	0.5	0.5	0	0	1	1	0	1	0
Nusplingen Fm	Germany	Jurassic	0.5	0	0	0.5	0.5	0.5	0	0	1	1	0	1	0
Aitou Fm	Lebanon	Cretaceous	1	0	0	0.5	0.5	0.5	0	0	1	0	0	1	0
Milna Fm, Hvar	Croatia	Cretaceous	1	0	0	0.5	0.5	0	0	0	1	0	0	1	0
Fm Mte Post-Pesc	Italy	Palaeog	0.5	0	0	1	0	0.5	0	0	1	0	0	1	0

For the literature sources, see text and Table 2. Coding: Marine basin size—0: < 1 km^2^; 0.5: 1 to 10 km^2^; 1: > 10 km^2^. Facies—0: limestone; 1: clastics. Palaeolatitude—0: tropical; 1: moderate. Thickness—0: < 1 m; 0.5: 1 to 10 m; 1: > 10 m. Duration—0: < 1 Ma; 1: 0.5 to 10 Ma; 1: > 10 Ma. Sea level—0: < 10 m; 0.5: 10 to 100 m; 1: > 100 m. Sediment structures—0: lamination; 1: ripple marks. Pyrite—0: absent; 1: present. Fauna—0: invert. 1: vert. (common). Traces- 0: absent; 1: present. Infauna—0: absent; 1: present. Epibenthos—0: absent; 1: present. Pelagics (macro)—0: nekton; 1: plankton (dominant). Death marches—0: absent; 1: present. Landing marks—0: absent; 1: present

*Thylacoc. L* Thylacocephalan Layer, Famennian, Morocco, *HBS* Hangenberg Black Shale, end Devonian, *Palaeog* Palaeogene

**Table 2:** Coding for the comparison of conservation deposits based on characteristics supposed by Seilacher et al. (1985), using data and modifications by Frey et al. (2019)

Name	Death marches	Landing marks	Soft parts	Cuticles	Articulation	Life position	Roll marks	Alignmennt	Aragonite	Pyrite steinkerns	Concretions	Deformation	Carbonization	Phosphatization
Ediacara	0	0	0	0	0	0	0	1	0	0	0	0	0	0
Maotianshan Shales	0	0	1	1	1	0	0	0	0	0	0	1	0	0
Burgess Shale	0	0	1	1	1	0	0	1	0	0	0	1	0	0
Fezouata Fm	0	0	1	1	1	0	0	0	0	0	0	1	0	0
Hunsrück Shale	1	0	1	1	1	0	0	1	0	1	0	1	0	1
Gogo Fm	0	0	1	1	1	0	0	0	0	0	1	0	0	1
Thylacoceph. Layer	0	0	1	1	1	0	0	0	0	1	1	1	0	1
Madene HBS	0	0	1	1	1	0	0	0	0	0	0	1	1	1
Bear Gulch Lst	0	0	1	1	1	0	0	0	0	0	0	1	1	1
Francis Creek Shale	1	0	1	1	1	0	0	0	0	0	1	1	1	1
Lower lst, Thaynes Gp	0	0	1	1	1	0	0	0	0	0	0	1	1	1
Wordie Creek Fm	0	0	0	0	1	0	0	0	0	0	1	1	0	0
Middle Sakamena Fm	0	0	0	0	1	0	0	0	0	0	1	1	0	0
Candelaria Fm	0	0	0	0	1	0	0	0	0	0	1	1	0	0
Osawa Fm	0	0	0	1	1	0	0	0	0	0	0	1	0	0
Nanlinghu Fm	0	0	0	0	1	0	0	0	0	0	0	1	0	0
Jialingjiang Fm	0	0	0	0	1	0	0	0	0	0	0	1	0	0
Sulphur Mt. Fm	0	0	0	0	1	0	0	0	0	0	0	1	0	0
Vikinghøgda Fm	0	0	0	0	1	0	0	0	0	0	1	0	0	0
MSG, Besano Fm	0	0	1	1	1	0	0	0	0	0	0	1	1	1
MSG, Cava inferiore	0	0	0	0	1	0	0	0	0	0	0	1	0	0
MSG, Cava superiore	0	0	1	1	1	0	0	0	0	0	0	1	1	1
MSG, Cassina	0	0	0	1	1	0	0	0	0	0	0	1	1	0
Kalkschieferzone	0	0	0	1	1	0	0	0	0	0	0	1	1	0
Prosanto Fm	0	0	0	1	1	0	0	0	0	0	0	1	0	0
Partnach Fm	0	0	1	0	1	0	0	0	0	0	0	1	0	0
Prida Fm	0	0	0	0	1	0	0	0	0	0	1	1	0	0
Botneheia Fm	0	0	0	0	1	0	0	0	0	0	0	1	0	0
Guanling Fm Luoping	0	0	0	1	1	0	0	0	0	0	1	1	1	0
Guanling Fm Panxian	0	0	0	0	1	0	0	0	0	0	1	1	0	0
Falang Fm Xingyi	0	0	0	0	1	0	0	0	0	0	0	1	0	0
Xiaowa Fm Guanling	0	0	0	0	1	0	0	0	0	0	0	1	1	0
Calcare di Zorzino	0	0	0	1	1	0	0	0	0	0	0	1	0	0
Lunz Fm	0	0	1	1	1	0	0	0	1	0	0	1	1	1
Seefeld Mb	0	0	1	1	1	0	0	1	0	0	0	1	1	1
Psilonotenton Fm	0	0	0	0	1	0	0	0	0	0	0	0	0	0
Posidonienschiefer Fm	0	0	1	1	1	0	0	1	0	1	1	1	1	1
Oxford Clay Fm	0	0	1	1	1	0	0	0	1	0	0	1	0	1
Terres noires, La Voulte	0	0	1	1	1	0	0	0	0	1	1	1	0	1
Altmühltal Fm	1	1	1	1	1	0	1	1	0	0	0	1	0	1
Painten Fm	1	1	1	1	1	0	1	1	0	0	0	1	1	1
Nusplingen Fm	1	1	1	1	1	0	0	1	0	0	0	1	1	1
Aitou Fm	0	0	1	1	1	0	0	1	0	0	0	1	1	1
Milna Fm, Hvar	0	0	1	1	1	0	0	1	0	0	0	1	1	1
Fm del Monte Postale-Pesciara	0	0	1	1	1	0	0	0	0	0	0	1	1	1

Name	Pyritization	Clay minerals	Silicification	Obrution	Stagnation	Bacterial/algal	Source
Ediacara	0	0	0	0	0	1	Selden & Nudds, 2012
Maotianshan Shales	1	1	0	0	1	0	Xian-Guang et al. 2003
Burgess Shale	0	0	0	1	1	0	Briggs et al., 1994
Fezouata Fm	1	1	0	1	0	0	van Roy et al., 2010, Martin et al., 2016
Hunsrück Shale	1	0	0	1	1	0	Bartels et al., 1998
Gogo Fm	1	0	0	0	1	0	Long & Trinajstic, 1982
Thylacoceph. Layer	1	0	1	0	1	0	This paper
Madene HBS	0	0	0	0	1	0	This paper
Bear Gulch Lst	0	0	0	1	0	0	Grogan & Lund, 2002
Francis Creek Shale	0	0	0	1	1	0	Selden & Nudds, 2012
Lower lst, Thaynes Gp	0	0	0	0	1	0	Brayard et al., 2017
Wordie Creek Fm	0	0	0	0	1	0	Brinkmann et al., 2010
Middle Sakamena Fm	0	0	0	0	1	0	Kogan & Romano, 2016
Candelaria Fm	0	0	0	0	1	0	Ware et al., 2011
Osawa Fm	0	0	0	0	1	0	Ehiro, 2022
Nanlinghu Fm	0	0	0	0	1	0	Du et al., 2023
Jialingjiang Fm	0	0	0	0	1	0	Li & Liu, 2020
Sulphur Mt. Fm	0	0	0	0	1	0	Neuman, 2015
Vikinghøgda Fm	0	0	1	0	1	0	Hurum et al., 2018
MSG, Besano Fm	1	0	0	0	1	0	Etter, 2002a
MSG, Cava inferiore	0	0	0	0	1	0	Furrer, 2003
MSG, Cava superiore	0	0	0	0	1	0	Furrer, 2003
MSG, Cassina	0	0	0	0	1	0	Stockar, 2010
Kalkschieferzone	0	0	0	0	1	0	Furrer, 2003
Prosanto Fm	0	0	0	0	1	0	Scheyer et al., 2017
Partnach Fm	0	0	0	0	1	0	Tichy, 1998
Prida Fm	0	0	0	0	1	0	Sander et al., 1994, 2021
Botneheia Fm	1	0	0	0	1	0	Engelschiøn et al., 2023
Guanling Fm Luoping	0	0	0	0	1	1	Hu et al., 2011
Guanling Fm Panxian	0	0	0	0	1	0	Jiang et al., 2009
Falang Fm Xingyi	0	0	0	0	1	0	Lu et al., 2018
Xiaowa Fm Guanling	0	0	0	0	1	0	Wang et al., 2008
Calcare di Zorzino	0	0	0	0	1	0	Tintori, 1992
Lunz Fm	0	0	0	0	1	0	Lukeneder & Lukeneder, 2021, 2022, 2023
Seefeld Mb	0	0	0	0	1	0	Hornung et al., 2019
Psilonotenton Fm	0	0	0	1	0	0	Seilacher et al., 1985
Posidonienschiefer Fm	1	0	0	0	1	0	Röhl et al., 2001, 2002
Oxford Clay Fm	0	0	0	0	1	0	Wilby et al., 2008
Terres noires, La Voulte	1	0	0	1	1	0	Etter, 2002b; Charbonnier, 2009
Altmühltal Fm	0	0	0	0	1	1	Arratia et al., 2015
Painten Fm	0	0	0	0	1	1	Arratia et al., 2015
Nusplingen Fm	0	0	0	0	1	1	Dietl & Schweigert, 2001
Aitou Fm	0	0	0	0	1	1	Forey et al., 2003
Milna Fm, Hvar	0	0	0	0	0	1	Hemleben & Freels, 1977
Fm del Monte Postale-Pesciara	0	0	0	0	1	0	Tang, 2002

Coding: for all characteristics 0: absent and 1: present

Since the various subunits of the Anisian and Ladinian strata at Monte San Giorgio bear different faunas, we discriminated between the Besano Formation, and the younger units Cava inferiore, Cava superiore, Cassina beds as well as the Kalkschieferzone (e.g., Stockar, 2010). For the Middle Triassic, we added several Chinese localities, which were highlighted as Konservat-Lagerstätten by Benton et al. (2013), being aware that there are several other important localities with articulated vertebrates in China. We assembled data for the localities Luoping, Yunnan (Guanling Fm; Hu et al., 2011), Panxian, Guizhou (Guanling Fm; Jiang et al., 2009), and Xingyi, Guizhou (Falang Fm; Lu et al., 2018). We complemented that list with some European Lagerstätten such as the Swiss Ducanfurgga (Prosanto Fm; Scheyer et al., 2017), which yielded some amazing vertebrate fossils recently (Cavin et al., 2017a, 2017b; Ferrante et al., 2023; Scheyer et al., 2017). Furthermore, we included data from the conservation deposits of the Prida Formation of Fossil Hill, Nevada (Kelley et al., 2016; Sander et al., 1994, 2021), Edgeøya, Svalbard (Botneheia Fm; Engelschiøn et al., 2023; Kear et al., 2016) and the Eosauropterygia-rich strata of the Gailtal in Carinthia (Partnach Fm; e.g., Rieppel, 1994; Tichy, 1998; Zapfe & König, 1980).

As far as the Late Triassic is concerned, we now included the Xiaowa Formation of Guanling, China (Jiang et al., 2005) as well as three European Lagerstätten. Austria has important conservation deposits of Late Triassic age, which received more attention recently. These are the Lunz Formation with the Polzberg biota (Lukeneder & Lukeneder, 2021, 2022, 2023) and the Seefeld Member of Wiestal (Hornung et al., 2019). From Italy, we included the Calcare di Zorzino (Tintori, 1992), which is renowned for the oldest pterosaurs and its splendid fish fauna. Of course, there are numerous more Triassic localities worldwide that yielded excellent fossils; a more comprehensive list was assembled by Brinkmann et al. (2010), but even this list needs an update.

As in Frey et al. (2019), we included the data of these 45 Lagerstätten listed in Tables 1 and 2 in principal component analyses on the variance–covariance matrix in PAST (Hammer et al., 2001). Eigenvalues are listed in Table 3. We then assembled data of the faunal composition and rough abundance estimates of the Triassic Lagerstätten in Table 4 with the eigenvalues in Table 5. We included rough estimates of relative abundances of most organism groups with a focus on eukaryotes, i.e. invertebrates are also included (for cephalopods, see, e.g., Airaghi, 1911; Rieber, 1969, 1970, 1973, 1974; Pieroni, 2022; Pohle et al. in press). Finer estimates of abundances can currently not be made since quantitative data are not available at the same precision for all localities and strata included in this analysis. We then ran another principal component analysis in PAST to compare the relative faunal composition of the Triassic sites (eigenvalues in Table 5). Additionally, we carried out hierarchical cluster analyses (paired group, Ward’s method) using the same datasets. The layout of all biplots was made with CorelDraw X8.

**Table 3:** Eigenvalues and variance of the principal components used for the comparison of conservation deposits

PC	Eigenvalue	% Variance
1	0.964394	22.386
2	0.698364	16.211
3	0.470158	10.913
4	0.324177	7.5249
5	0.252938	5.8713
6	0.239602	5.5617
7	0.189823	4.4062
8	0.163697	3.7998
9	0.144394	3.3517
10	0.123463	2.8658

For the matrix, see Tables 1 and 2

**Table 4:** Standardized coding for the comparison of Triassic conservation deposits based on faunal composition

Name	Other organisms	Algae	Higher plants	Ammonoids	Other cephalopods	Bivalves	Other benthic molluscs	Arthropods	Crinoids	Other echinoderms	Agnathans and conodonts	Chondrichthyans	Actinopterygians	Sarcopterygians
Lower lst, Thaynes Gp	1	1	0	0.5	1	0.5	1	1	0.5	1	0.5	0.5	0.5	0
Wordie Creek Fm	0	0	0	1	0	1	0	0	0	0	0.5	0.5	1	1
Middle Sakamena Fm	0	0	0	0.5	0	0.5	0	0.5	0	0	0	0.5	1	1
Candelaria Fm	0	0	0	1	0.5	1	0	0	0	0	0.5	0	0.5	0
Osawa Fm	0	0	0.5	1	0.5	0	0	0.5	0	0	0.5	0.5	0.5	0
Nanlinghu Fm	0.5	0	1	0.5	0	0.5	0.5	0.5	0	0	0.5	0	1	0
Sulphur Mt. Fm	0	0	0	0.5	0	0.5	0	0	0	0	0.5	0.5	1	1
Jialing- jiang Fm	0	0	0	1	0	1	0	0	0	0	0.5	0	1	0
Viking- høgda Fm	0	0.5	0.5	1	0	1	1	0	0	0	1	1	1	1
MSG, Besano Fm	0	0.5	0.5	0.5	1	0.5	1	0.5	0.5	1	0.5	0.5	1	1
MSG, Cava inferiore	0	0.5	0	0	0	0.5	0.5	0	0	0	0	0	1	0
MSG, Cava superiore	0	0.5	0	0.5	0	0.5	0.5	0	0	0.5	0	0	1	0
MSG, Cassina	0.5	0.5	1	0	0	0	0	0	0	0	0.5	0	1	0
Kalkschieferzone	0.5	0.5	0.5	0	0	0	0.5	1	0	0	0	0	1	0
Prosanto Fm	0	0	0.5	0	0	0	0	0	0	0	0.5	0	1	1
Partnach Fm	0	0	0	0.5	0.5	0.5	0	0	0	0	0	0	0	0
Prida Fm	0	0	0	0	1	1	0	0	0	0	1	0.5	0.5	1
Botneheia Fm	0	0	0	0.5	0	1	0	0	0	0	0.5	1	1	1
Guanling Fm Luoping	1	0	0.5	0.5	1	1	1	1	0.5	0	1	0	1	1
Guanling Fm Panxian	0	0	0.5	0.5	0	1	0	0	0	0	0.5	0	1	0
Falang Fm Xingyi	1	0	0.5	1	0	0.5	0	1	0	0	0.5	0	1	0
Xiaowa Fm Guanling	1	0	1	1	1	1	1	0.5	1	0	0.5	0.5	0.5	0
Calcare di Zorzino	0.5	0	0.5	0	0	1	0.5	0.5	0.5	0.5	0	0.5	1	0
Lunz Fm	1	1	0.5	1	1	1	1	0.5	0	1	0.5	0.5	1	1
Seefeld Mb	0	0	0	0	0	0	0	0.5	0	0	0	0	1	1

Name	Ichthyosaurs	Thalattosaurs	Eosauropterygians	Placodonts	Tanystrophaeids s.l	Other vertebrates	
Lower lst, Thaynes Gp	0	0	0	0	0	0	Brayard et al., 2017
Wordie Creek Fm	0	0	0	0	0	0	Brinkmann et al., 2010; Kear et al., 2016
Middle Sakamena Fm	0	0	0	0	0	1	Kogan & Romano, 2016
Candelaria Fm	0	0	0	0	0	0	Ware et al., 2011
Osawa Fm	0.5	0	0	0	0	0	Ehiro, 2022
Nanlinghu Fm	1	0	0.5	0	0	0	Du et al., 2023
Sulphur Mt. Fm	1	0.5	0	0	0	0	Neuman, 2015
Jialing- jiang Fm	0	0	0.5	0	0	1	Li & Liu, 2020
Viking- høgda Fm	1	0	0	0	0	0.5	Hurum et al., 2018
MSG, Besano Fm	1	0.5	1	0.5	0.5	0.5	Etter, 2002a
MSG, Cava inferiore	0	0	0.5	0	0.5	0	Furrer, 2003
MSG, Cava superiore	0	0	0.5	0	0	0	Furrer, 2003
MSG, Cassina	0	0	0.5	0	0.5	0	Stockar, 2010
Kalkschieferzone	0	0	0.5	0	0	0	Furrer, 1995, 2003
Prosanto Fm	0	0	0.5	0	0.5	0	Scheyer et al., 2017
Partnach Fm	0	0	1	0	0.5	0	Tichy, 1998
Prida Fm	1	0	0	0	0	0	Sander et al., 1994
Botneheia Fm	1	0	0	0	0	0	Brinkmann et al., 2010; Kear et al., 2016
Guanling Fm Luoping	1	0	1	0	0	1	Benton et al. 2013
Guanling Fm Panxian	1	0	1	0.5	0.5	1	Benton et al. 2013
Falang Fm Xingyi	0.5	0.5	1	0.5	0.5	0	Benton et al. 2013
Xiaowa Fm Guanling	1	1	0	0.5	0	0.5	Benton et al. 2013
Calcare di Zorzino	0	0.5	0	0.5	0.5	0.5	Tintori, 1992
Lunz Fm	0	0	0	0	0	0	Lukeneder and Lukeneder 2021, 2022, 2023
Seefeld Mb	0	0	0	0	0	0	Hornung et al., 2019

For the literature sources, see text and Table 2. Coding: 0: absent or rare; 0.5: moderately common; 1: relatively abundant. For abbreviations see caption of Table 2

**Table 5:** Eigenvalues and variance of the principal components used for the comparison of Triassic conservation deposits based on faunal composition

PC	Eigenvalue	% Variance
1	0.643425	24.364
2	0.470369	17.811
3	0.33971	12.864
4	0.233635	8.847
5	0.178979	6.7774
6	0.160213	6.0667
7	0.134371	5.0882
8	0.125886	4.7669
9	0.0854462	3.2356
10	0.0731675	2.7706

## Results

### Characteristics of the Konservatlagerstätten of Monte San Giorgio

Like several other Triassic conservation deposits, today’s Monte San Giorgio was located at the margin of the Tethys in a tropical latitude during the Middle Triassic (Fig. 6; see, e.g., Lu et al., 2018; Benton et al., 2013). Lithologically, the Besano Formation is dominated by light grey to dark grey dolomitized limestones and black shales (e.g., Arif et al., 2019; Bassani, 1886; Baumgartner et al., 2001; Bernasconi, 1994; Felber, 2006; Furrer, 1995, 2003, 2004; Röhl et al., 2001; Stockar et al., 2012). Most of the strata that yielded articulated vertebrate skeletons (sometimes with embryos or soft-tissue remains: Figs. 2B, 5) are thin-bedded and laminated black shales. In these respects, this Lagerstätte is quite similar to several of those from China (Guanling Fm of Luoping and Panxian, Falang Fm of Xingyi, Xiaowa Fm of Guanling; Benton et al., 2013). The fossiliferous Besano Formation is about 16 m thick and overlies the Salvatore Dolomite (Stockar, 2010). Above, the San Giorgio Dolomite and the Ladinian Meride Limestone follow; the latter contains further beds of Lagerstätten quality such as the Cava inferiore and superiore as well as the Cassina beds and the Kalkschieferzone (Stockar et al., 2010). Except for the somewhat more carbonatic Kalkschieferzone (hence the name), the other four units are dominated by finely laminated limestones and black shales (Stockar, 2010). Depending on the clay content, the fossils (including the bones) are flattened to varying degrees. This applies particularly to the articulated skeletons, although those preserved in carbonatic strata display three-dimensionally preserved bones, occasionally even undeformed. For a recent account of the fossil content with a focus on vertebrates, see Rieppel (2019).

**Fig. 7:**
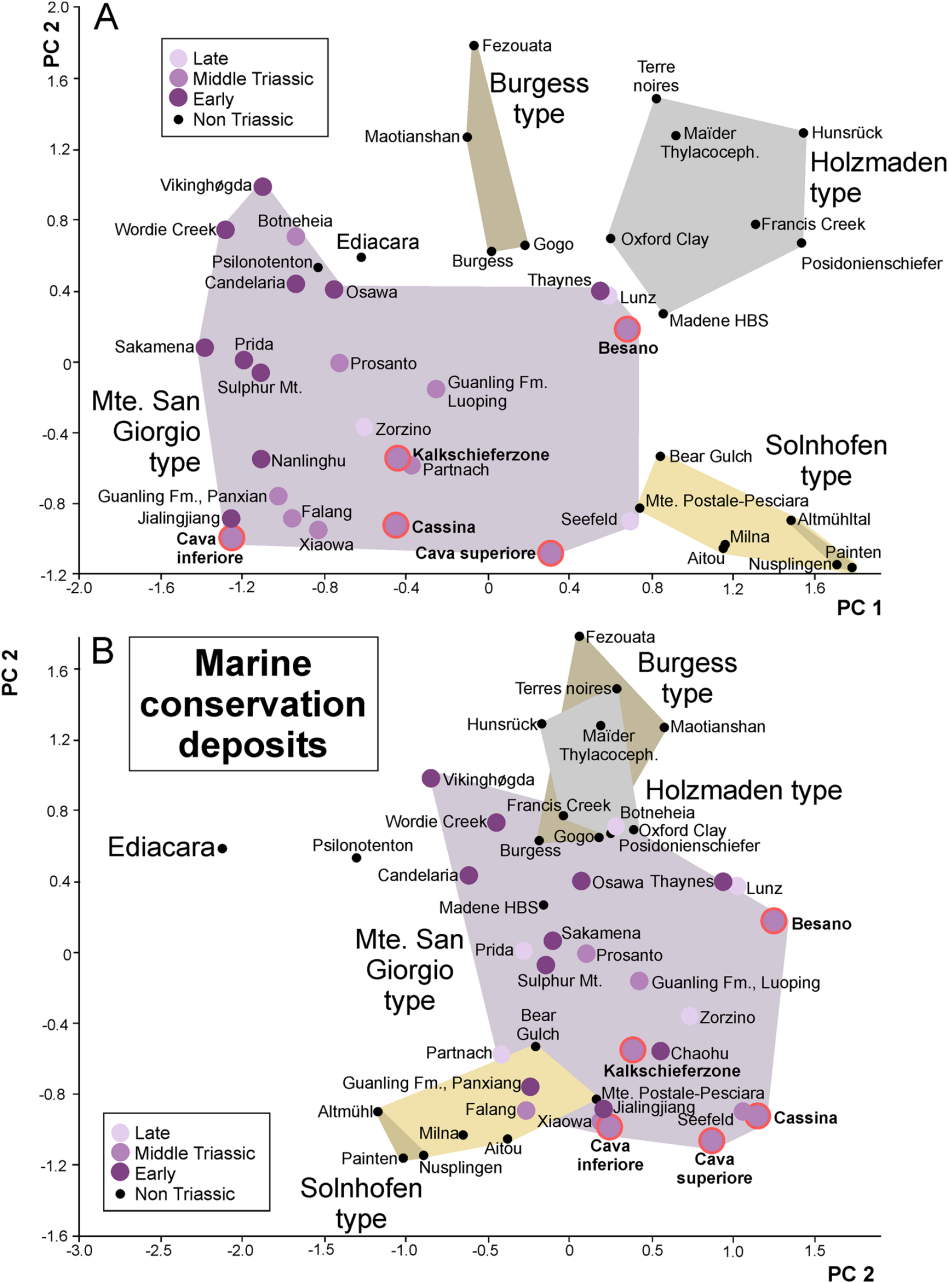
Classification of marine conservation deposits using a variation–covariation principal components analysis and the characterization of the Besano Formation of Monte San Giorgio (red margin). Data are listed in Tables 1 and 2. Modified after Frey et al. (2019). Note that among Triassic deposits, Monte San Giorgio, the Paris biota (Thaynes Fm) and Polzberg (Lunz Fm) are the only ones plotting close to the classic Black Shale Lagerstätten in A. **A** PCA 1 and 2; **B** PCA 2 and 3

**Fig. 8:**
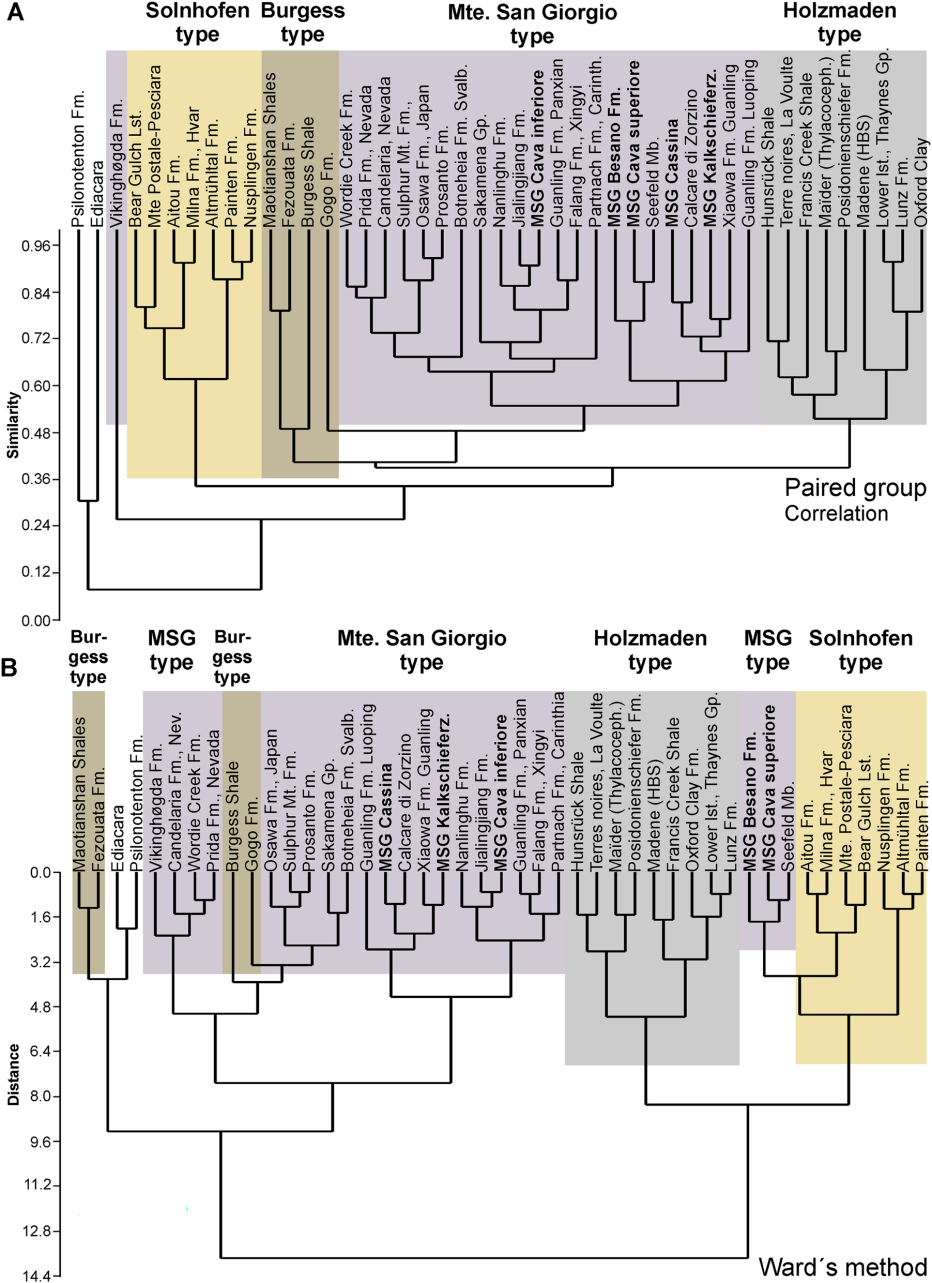
Classification of marine conservation deposits using cluster analyses. **A** paired groups. **B** Ward’s method. Note how in both cases, the Solnhofen and Holzmaden type Lagerstätten cluster well

Interestingly, the Besano Formation of Monte San Giorgio plotted somewhat separately from the other black shale deposits compared to the analysis of Frey et al. (2019) and in a different region than the other fossiliferous units at Monte San Giorgio (Fig. 7). In one of the cluster analyses (Fig. 8B), the Besano Formation formed a cluster with the Seefeld Member of Wiestal and the Cava superiore, while the other three units of Monte San Giorgio fell in a different place. In the PCA, the data points of all the Chinese Lagerstätten plot in the vicinity of the Cava inferiore and superiore, the Cassina beds and the Kalkschieferzone; this pattern was also found in the cluster analyses of Fig. 8. The data points of the classic black shales such as Holzmaden (Posidonia shale, Toarcian, Jurassic; Röhl et al., 2002), Bundenbach (Hunsrück slate, Emsian, Devonian; Bartels et al., 1998; De Baets et al., 2013) or Christian Malford (Oxford Clay, Jurassic; Wilby et al., 2008) lie in their own cluster (grey in Figs. 7, 8). In the PCA, the data point of the Besano Formation plots at the margin of the field of the Monte San Giorgio type conservation deposits and more or less between the fields occupied by Solnhofen type and the Holzmaden type Lagerstätten. This was expected considering that in all these deposits, flattened but articulated vertebrate skeletons are common, phosphatized soft-tissues are preserved, and pyritization occurs, albeit to differing extends (very common in the Hunsrück slate and the Posidonia shale while relatively rare in the Besano Formation: Fig. 5). Importantly, the facies of Monte San Giorgio is intermediate given the fact that it contains both laminated limestones and black shales. Accordingly, the Kalkschieferzone and Cava inferiore plot closer to the Solnhofen type in Fig. 7B. Fossil preservation, faunal composition (lack or scarcity of benthos), sedimentary facies, and the palaeogeographical setting suggest at least episodically anoxic conditions (Röhl et al., 2001; Stockar et al., 2012).

### Triassic marine conservation deposits worldwide

**Fig. 9:**
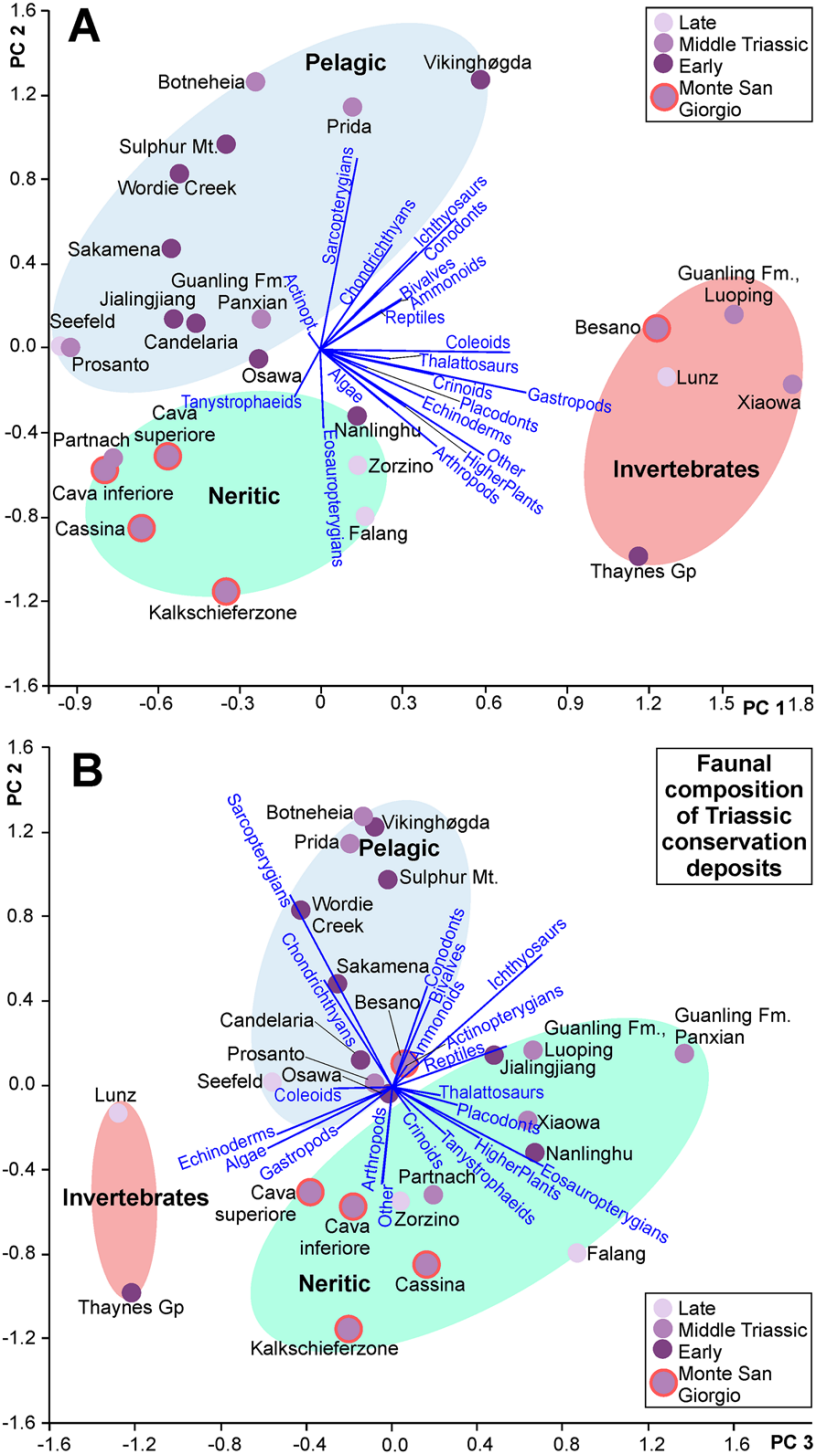
Classification of Triassic marine conservation deposits based on abundances of fossil groups using a variation–covariation principal components analysis and the characterization of the fossiliferous units of Monte San Giorgio (red margin). Data are listed in Table 4. Note the similarity in faunal composition of Monte San Giorgio and the Chinese Lagerstätten. The Paris biota (Thaynes Fm) and Polzberg (Lunz Fm) are special in being more invertebrate dominated. The fish localities overlap with those with abundant reptiles (e.g., Ducan). **A** PCA 1 and 2; **B** PCA 2 and 3. Reptiles in the graph stands for other reptile groups

**Fig. 10:**
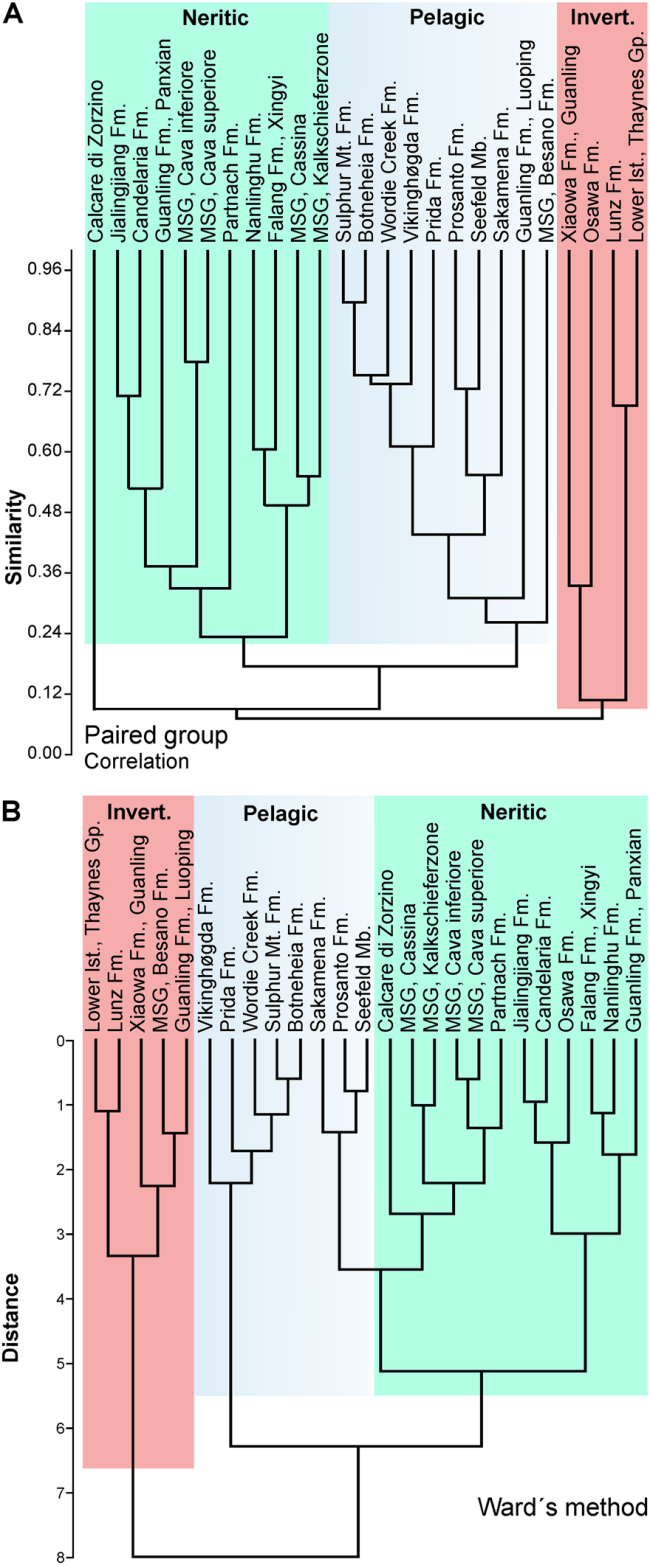
Classification of Triassic marine conservation deposits based on abundances of fossil groups using a hierarchical cluster analysis and the characterization of the fossiliferous units of Monte San Giorgio (red margin). Data are listed in Table 4

As mentioned above, numerous Triassic localities with preservation modes similar to those known from the fossiliferous beds at Monte San Giorgio have been discovered in South China in the last decades (e.g., Benton et al., 2013; Li, 2006; Lu et al., 2018). Some are from the Early Triassic (Chaoxian, Nanzhang, Wuming, Yuan’an), several from the Middle Triassic (Dingxiao, Guiyang, Fuyuan, Luoping, Luxi, Panxian, Qingzhen, Renhuai, Xingyi) and at least three from the Late Triassic (Guanling, Nylamu, Tingri). Although fossil preservation surprisingly often resembles that of the Besano Formation of Monte San Giorgio (Xiaowa Fm of Guanling, Daye Fm of Guiyang, Guanling Fm of Panxian, Falang Fm of Xingyi), the overall taxonomic composition is similar but differs in key aspects (Table 4, Figs. 9, 10). Also, the respective abundances of the various vertebrate groups are not identical (Benton et al., 2013). The upper part of the Besano Formation yielded many fishes, *Neusticosaurus* (a pachypleurosaurid eosauropterygian) and *Mixosaurus* (an ichthyosaur), while some of the Chinese localities like the Xiaowa Fm of Guanling (Benton et al., 2013; Liu et al., 2013; Rieppel, 2019; Wang et al., 2008) are, e.g., rich in thalattosaurs. Furthermore, the huge pseudoplanktonic crinoid colonies of *Traumatocrinus* present at Guanling (Hagdorn et al., 2007) are unknown from the Alpine occurrences but are comparable to the driftwood crinoid colonies of *Moroccocrinus* of Late Devonian age from Morocco (Frey et al., 2018, 2019; Klug et al., 2003) and *Seirocrinus* found in the Jurassic Posidonia shale (e.g., Hess, 1999).

In addition to the Chinese occurrences, well comparable conservation deposits of Triassic age with black shale characteristics in a broad sense (i.e. dark sediment, complete skeletons, poor in benthos, rich in clay) have been recognized in many other places. Examples for such deposits have been documented from Europe (eastern Switzerland, Ducan: Furrer, 2004; western Austria: Zapfe & König, 1980; Wachtler & Perner, 2018; Italy: Stefani et al., 1991; Slovakia: Čerňanský et al., 2018; Slovenia: Hitij et al., 2010), Asia (Spiti, India: Romano et al., 2016; Myanmar: San et al., 2019), and North America (Idaho: Brayard et al., 2017; Nevada: Romano et al., 2019).

Our principal components analysis shown in Fig. 9 allows a classification according to the abundance of (i) more pelagic groups such as ammonoids, fishes and ichthyosaurs; (ii) more neritic groups such as eosauropterygians or tanystrophaeids or (iii) generally invertebrates. Remarkably, the data points of Cava inferiore, Cava superiore, Cassina Beds and the Kalkschieferzone again plotted closer to each other than to the point of the Besano Formation. This pattern is also seen in the cluster analysis in Fig. 10. When regarding the PC1/PC2-plot, the Besano Formation-point is in the field of Lagerstätten with abundant invertebrates, while when regarding PC2 and PC3, it falls quite central in the neritic groups/ reptile-dominated field, possibly due to the abundance of eosauropterygians, thalattosaurs and placodonts. The other four units of Monte San Giorgio fall in their own field in the PC2/PC3-plot with other neritic animal-dominated Lagerstätten.

## Discussion

In our principal component analysis presented in Fig. 7, it is remarkable how well especially the platy limestones (Solnhofen type) separate from all other marine Lagerstätten, at least in PC1 and PC2. The German localities plot even closer together within this field (lower right corner in Fig. 7A). They formed their own cluster in both cluster analyses (Fig. 8). The Palaeozoic localities of the Cambrian and Ordovician also fell in a quite well delineated field, which comprises all of the Burgess type both in the PCA and in one of the cluster analyses (Fig. 8A). Likewise, the classic black shale occurrences (Holzmaden type) plot close to each other in the PCA and in both cluster analyses (Fig. 8), while the Monte San Giorgio type organic-rich deposits are somewhat scattered in the PCA (Fig. 7), occupying a large but separate field, thus suggesting a somewhat variable palaeoenvironment. Remarkably, this includes occurrences of Early, Middle and Late Triassic age. It is worth mentioning that this pattern remained stable even after several changes in the matrix. Unsurprisingly, the localities of the Chinese Triassic included here lie quite close to each other in the PCA and the cluster analyses. It is also noteworthy that, in the PC2/PC3-plot (Fig. 7B), the field of the Triassic Monte San Giorgio type Lagerstätten overlaps the fields comprising the black shales deposits of the Holzmaden and the Burgess type as well as the platy limestones of the Solnhofen type. The data points of the only Proterozoic Lagerstätten Ediacara and the obrution deposit Gmünd (Psilonotenton) usually fall more or less separate from the other points both in the PCAs and the cluster analyses.

The peculiar grouping of Monte San Giorgio type Lagerstätten (Figs. 7A, 8A, 9B), is remarkable. We suggest that the highly unusual palaeoecological conditions needed to produce the sedimentary facies of these fossiliferous beds can be explained by the long-term effects of the Permian–Triassic boundary mass extinction (Benton, 2016; Burgess et al., 2014). This created a palaeoenvironment with low oxygen conditions, euxinia, and acidification (Galfetti et al., 2007; Goudemand et al., 2019; Payne et al., 2004, 2012; Romano et al., 2013) and other special conditions, that were unusually widespread throughout much of the Triassic. In turn, this is linked with low diversity benthos in many basins including a slow reef recovery (Benton, 2016).

In our second analysis, we focused on the faunal composition of the Monte San Giorgio type Lagerstätten. The Paris (Idaho; Thaynes Fm) and Polzberg biota (Austria; Lunz Fm) yield abundant and diverse invertebrates and plot near each other in both the PCA (Fig. 9) and the cluster analyses (Fig. 10). The other localities are distributed over the PCA-biplots according to whether their fauna is rather dominated by pelagic or neritic animals. A similar result was found in the cluster analyses, although the grouping differs somewhat (Fig. 10). In the PC1–PC2 plot, the Besano Formation of Monte San Giorgio is nested between the invertebrate dominated localities, while in the PC2–PC3 plot, it falls in the field with abundant thalattosaurs, placodonts and other more neritic animals, similar to some Chinese localities.

To some degree, the groupings correspond to our expectations, i.e. we knew that the localities of Madagascar (Sakamena Fm) and Ducanfurgga (Prosanto Fm) are fish-dominated while invertebrates are very common in the biotas of Polzberg (Lunz Fm) and Paris (Thaynes Fm). The question arises to what extent the results depend on the sampling effort and the quality of the documentation of discoveries. Particularly, the discovery of larger vertebrates may depend on larger excavations over longer time spans. Future excavations in other localities should optimally be bed-by-bed like many excavations at Monte San Giorgio and should document abundance data (specimen counts per bed per surface area or rock volume). Although the Besano Formation at Monte San Giorgio has become one Triassic marine conservation deposit out of many, it is the pioneer of this kind of deposit in the Triassic and will remain an important reference in the future. Accordingly, we consider it adequate to name this kind of Triassic conservation deposits ‘Monte San Giorgio type Lagerstätten’.

## Conclusions

The conservation deposits of Anisian and Ladinian age of Monte San Giorgio, comprising the Besano Formation, Cava inferiore, Cava superiore, Cassina Beds, and the Kalkschieferzone, represent some of the first black shale conservation deposits of Triassic age that were thoroughly studied. Now, after a century of excavations and more than a century of research, these deposits begin to enjoy global scientific recognition (e.g., Etter, 2002a; Rieppel, 2019), and continue to produce valuable new information about the palaeobiology and evolution of Triassic vertebrates today.

With this paper, we want to highlight the key role of the conservation deposits of Monte San Giorgio: comparable to the pioneer role of the Burgess Shale for the Cambrian Lagerstätten or Solnhofen for the Mesozoic platy limestones, we highlight the pioneer role of the Besano Formation in particular as the prototype for Triassic Lagerstätten. Our simple comparison of 45 Fossillagerstätten worldwide employing principal component and hierarchical cluster analyses of 32 traits based on the list of Seilacher et al. (1985) confirm that the Besano Formation of Monte San Giorgio Lagerstätte is remarkably similar to other Triassic black shale deposits including, e.g., those of the Swiss Ducanfurgga and the South China block. The Triassic black shale deposits demonstrably occupy their own field separate from the Burgess type black shales, Solnhofen type platy limestones or Holzmaden type black shales. Accordingly, we introduce the term Monte San Giorgio type black shales.

The separate position of the Monte San Giorgio type organic-rich sediments can be explained by the mix of laminated limestones and black shales and the scarcity of benthics, as well as the rise of several new groups such as important clades of marine reptiles. This is to some extent very likely an effect of the long-term ecological impact of the Permian–Triassic boundary mass extinction and the recovery of marine biotas.

Concerning the fossil content of the Monte San Giorgio type Lagerstätten, we found the three main groups ‘pelagic dominated’, ‘neritic dominated’ and ‘invertebrate dominated’. For some of the included Lagerstätten, the position in the PCA-plots and the cluster analyses was expected, for some others we suspect that over the years, more vertebrates and particularly reptiles may be discovered with longer or increased collecting/sampling activities. Accordingly, the respective position might change in the future.

## Data Availability

Specimens with PIMUZ numbers  are kept in the collection of the Palaeontological Institute of the University of Zurich. All data included in our analyses have been published previously and the sources are provided in Tables 2 and 4.
